# Color-Flow Doppler Detection of Partial Anomalous Pulmonary Venous Drainage in a Dog

**DOI:** 10.1016/j.case.2026.05.010

**Published:** 2026-07-13

**Authors:** Abigail T. Kaplan, Kailah M. Buchanan, Weihow Hsue

**Affiliations:** Department of Clinical Sciences, College of Veterinary Medicine, Cornell University, Ithaca, New York

**Keywords:** Atrial septal defect, Computed tomography angiography, Partial anomalous pulmonary venous connection, Sinus venosus, Veterinary

## Abstract

• PVeins enter both atria in anomalous pulmonary venous drainage.
• PAPVD can cause flow reversal on color-flow Doppler.
• Sinus venosus defects are often linked to anomalous pulmonary venous connections.

## Graphical abstract

**Figure:**
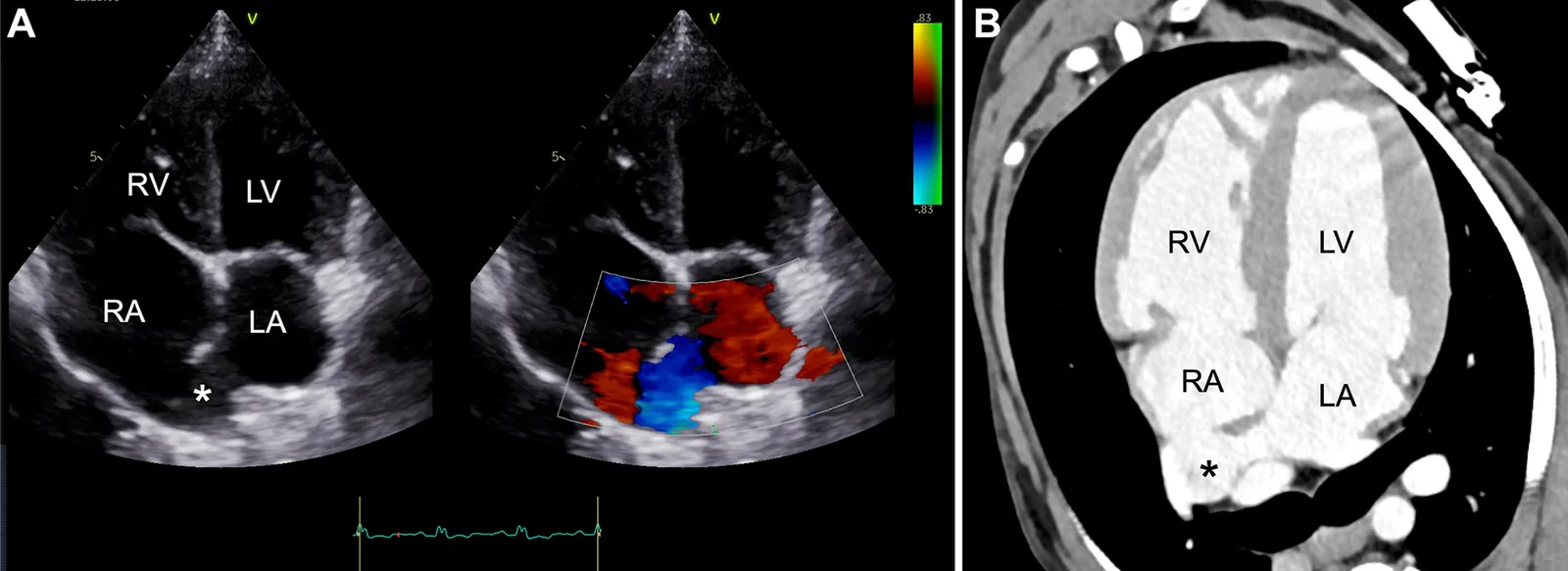

## Introduction

Left-to-right shunting at the pulmonary venous level can be categorized as anomalous connection or anomalous drainage.^1^ Partial anomalous pulmonary venous connection (PAPVC) refers to abnormal linking of some of the pulmonary veins (PVeins) to the right atrium (RA) or its tributaries without normal connection to the left atrium (LA), whereas total anomalous pulmonary venous connection involves all PVeins. In most cases, the terms “anomalous pulmonary venous connection” and “anomalous pulmonary venous return” are used interchangeably. In contrast, partial anomalous pulmonary venous drainage (PAPVD) occurs when some of the PVeins drain into the RA or its tributaries while retaining normal connection to the LA, whereas total anomalous pulmonary venous drainage involves all PVeins. This case report describes the initial suspicion of PAPVD in a young dog undergoing echocardiographic evaluation for a heart murmur and pulmonary valve (PV) stenosis.

## Case Presentation

A 2-year-old female spayed poodle mix was presented to the dermatology service of the university teaching hospital for chronic right-sided otitis externa. She also had a history of a heart murmur that had not been previously evaluated. Clinical signs were limited to pruritus and progressive pain affecting the right ear over the past year, with no other abnormalities reported. A grade IV/VI left basilar systolic murmur was auscultated on physical examination. Otoscopic examination of the right ear yielded gray-to-white purulent discharge and a dark object near the tympanic membrane. A foreign body within the right horizontal ear canal was suspected, and cardiology consultation was pursued in anticipation of general anesthesia for computed tomography (CT) and video otoscopy.

Transthoracic echocardiography (TTE) revealed moderate right atrial and right ventricular dilation with only mild right ventricular wall thickening (Figure 1A, Video 1). The tricuspid valve was normal in appearance, with mild tricuspid regurgitation (Figure 1B). Acoustic dropout was noted between a vessel and the RA on the right parasternal long-axis views; the vessel was deemed to be the right pulmonary artery based on the presence of systolic flow turbulence on color-flow Doppler (CFD) and the absence of flow between the 2 structures (Figure 2A). Nonturbulent flow originating from the dorsal aspect of the RA was also observed, although it was difficult to differentiate from caval flow (Figure 2B). The PV was mildly thickened and exhibited systolic doming, consistent with PV stenosis (Figure 3A), with trace pulmonary regurgitation. These findings accounted for the mild main pulmonary artery enlargement, moderate right pulmonary artery enlargement, and systolic flow turbulence within these vessels (Figure 3B). Pulmonary outflow velocity was increased at 3.67 m/s, corresponding to an estimated pressure gradient of 54 mm Hg using the modified Bernoulli equation. Most notably, CFD identified continuous flow reversal from the LA into the right cranial and/or right middle lobe PVein despite otherwise normal left heart structures (Figure 4, Video 2). Systematic interrogation in additional planes suggested a potential connection between the affected PVein and the roof of the RA; however, possible flow across the connection was inconsistently visualized, limiting definitive confirmation (Figure 5A, Video 3). An agitated saline study was declined by the dog's owner to avoid intravenous catheter placement.

**Figure 1:**
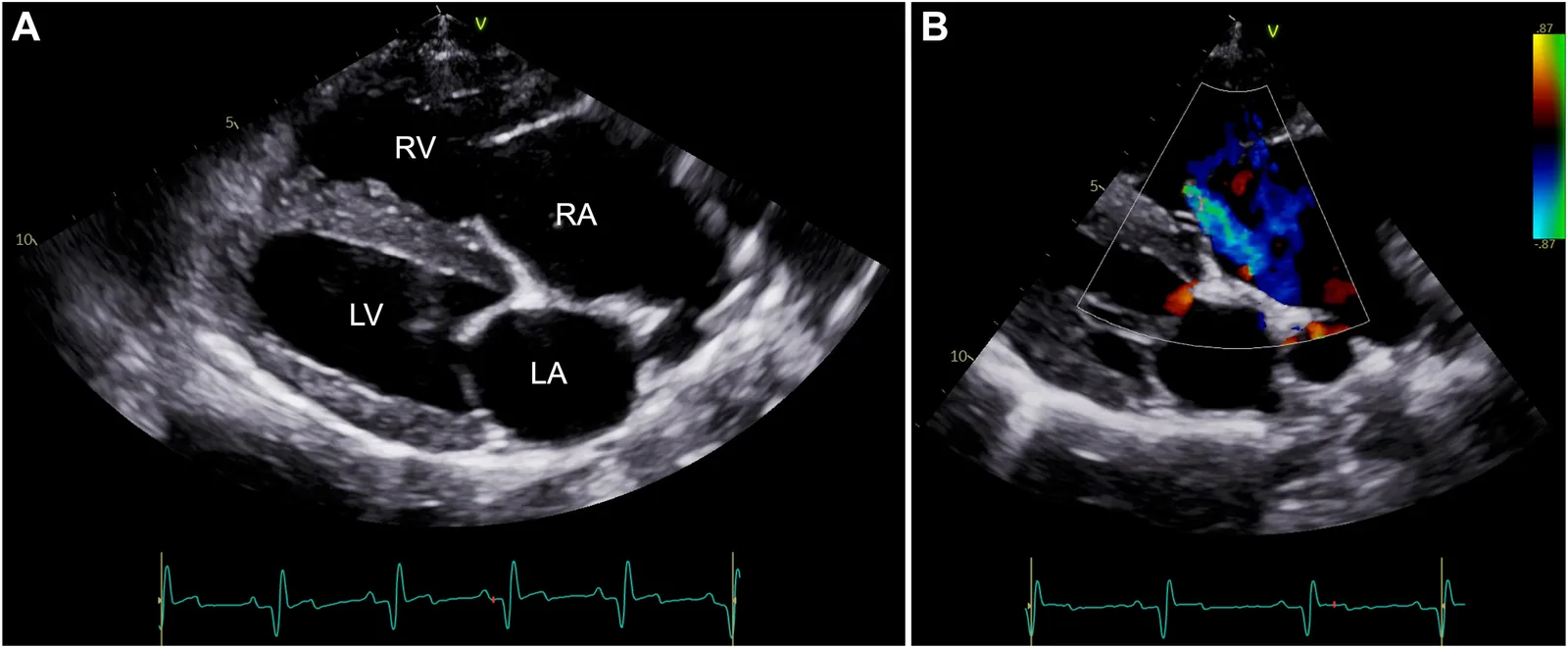
Two-dimensional TTE, right parasternal long-axis 4-chamber view, without **(A)** and with **(B)** CFD, demonstrates moderate dilation of both the RA and RV and mild tricuspid regurgitation. *LV*, Left ventricle; *RV*, right ventricle.

**Figure 2:**
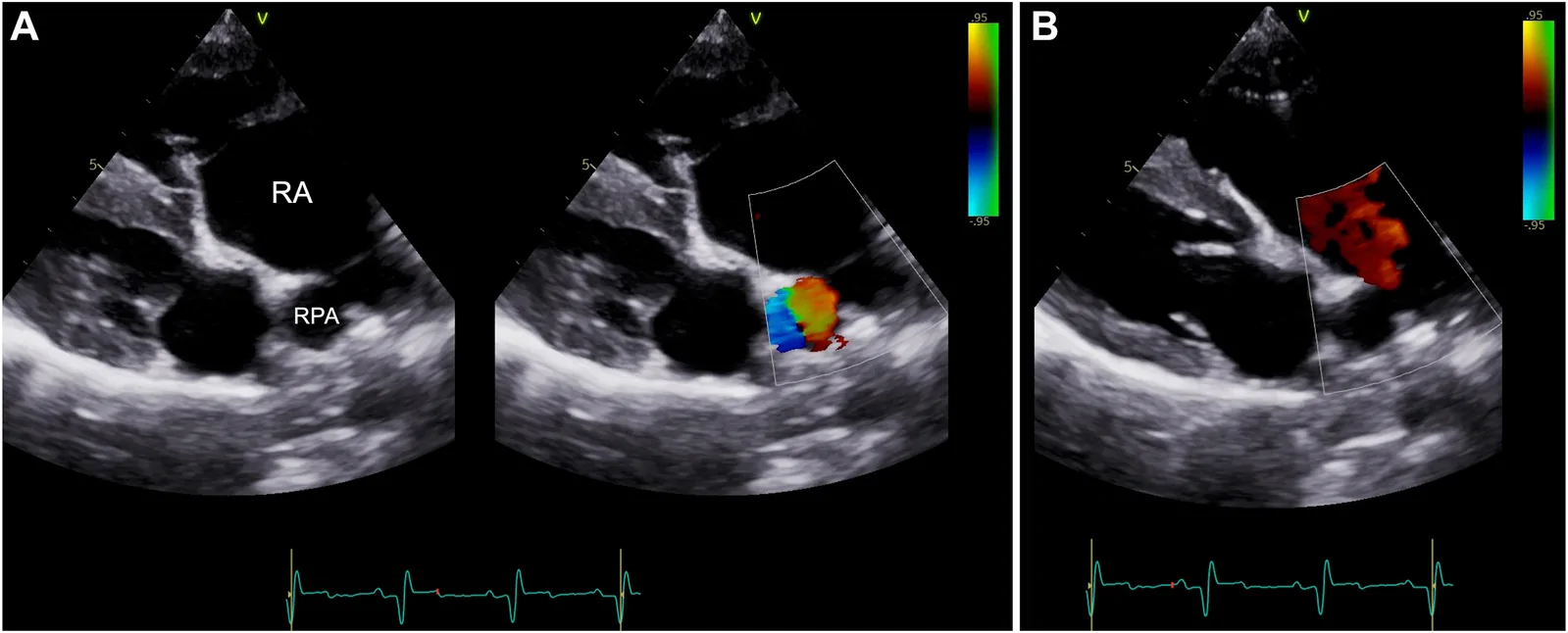
Two-dimensional TTE, right parasternal long-axis 4-chamber systolic **(A)** and diastolic **(B)** views, highlights structures surrounding the RA. During systole, simultaneous CFD imaging demonstrates flow turbulence in the RPA, without evidence of direct flow into the RA despite apparent acoustic dropout at the interface. During diastole, prominent flow entering the dorsal RA was also observed on CFD, although it could not be reliably distinguished from caval flow. *RPA*, Right pulmonary artery.

**Figure 3:**
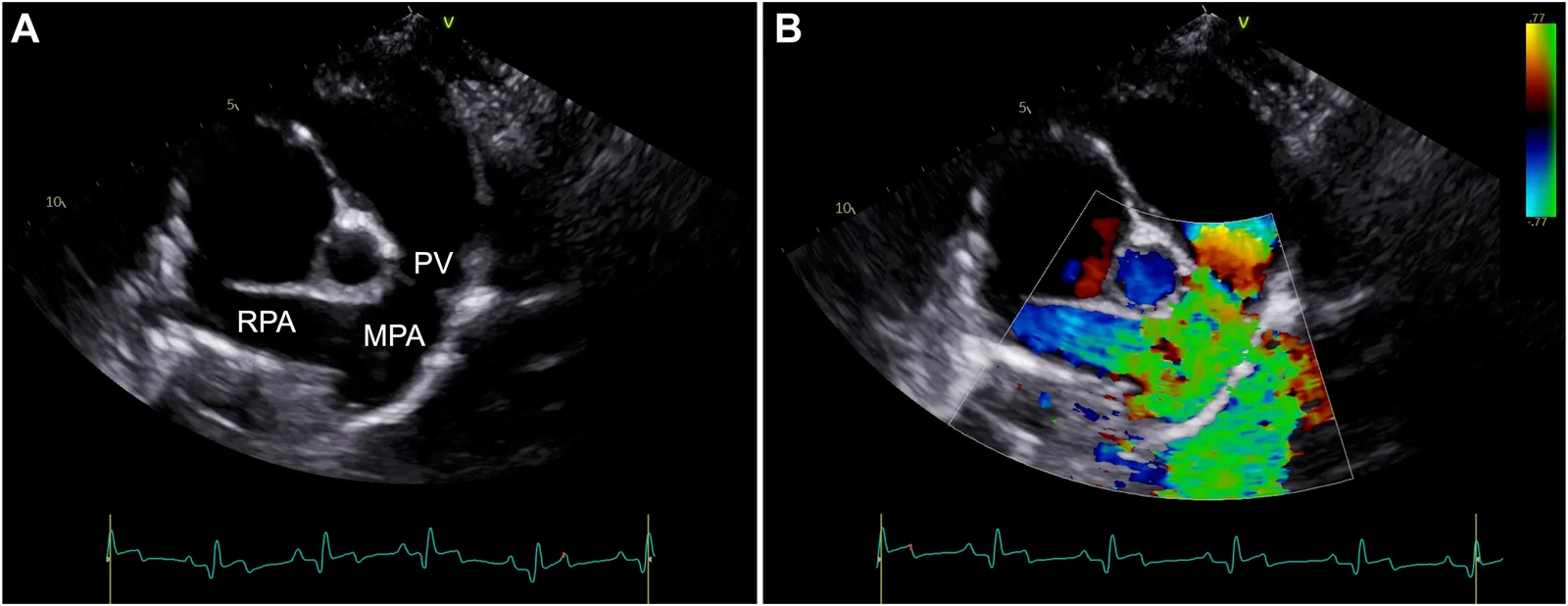
Two-dimensional TTE, right parasternal short-axis basilar view, without **(A)** and with **(B)** CFD, demonstrates PV stenosis. The PV is doming during systole, and systolic flow turbulence was observed beyond the PV in the enlarged MPA and RPA. *MPA*, Main pulmonary artery; *RPA*, right pulmonary artery.

**Figure 4:**
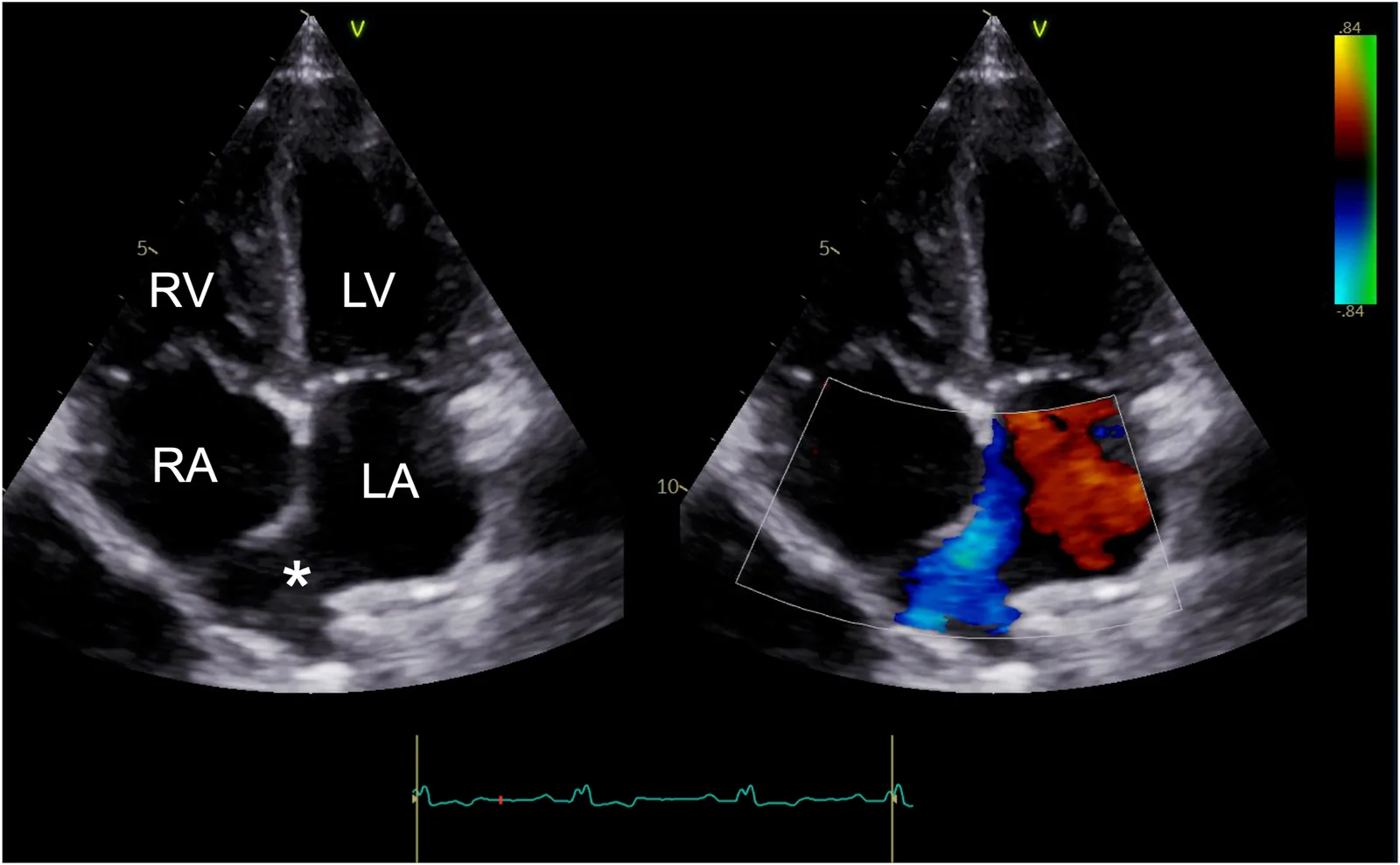
Two-dimensional TTE, left apical 4-chamber view with simultaneous CFD, demonstrates aberrant pulmonary venous flow. The LA and the right cranial and/or right middle lobe PVein (∗) are visualized, with continuous flow directed away from the LA into the right PVein. *LV*, Left ventricle; *RV*, right ventricle.

**Figure 5:**
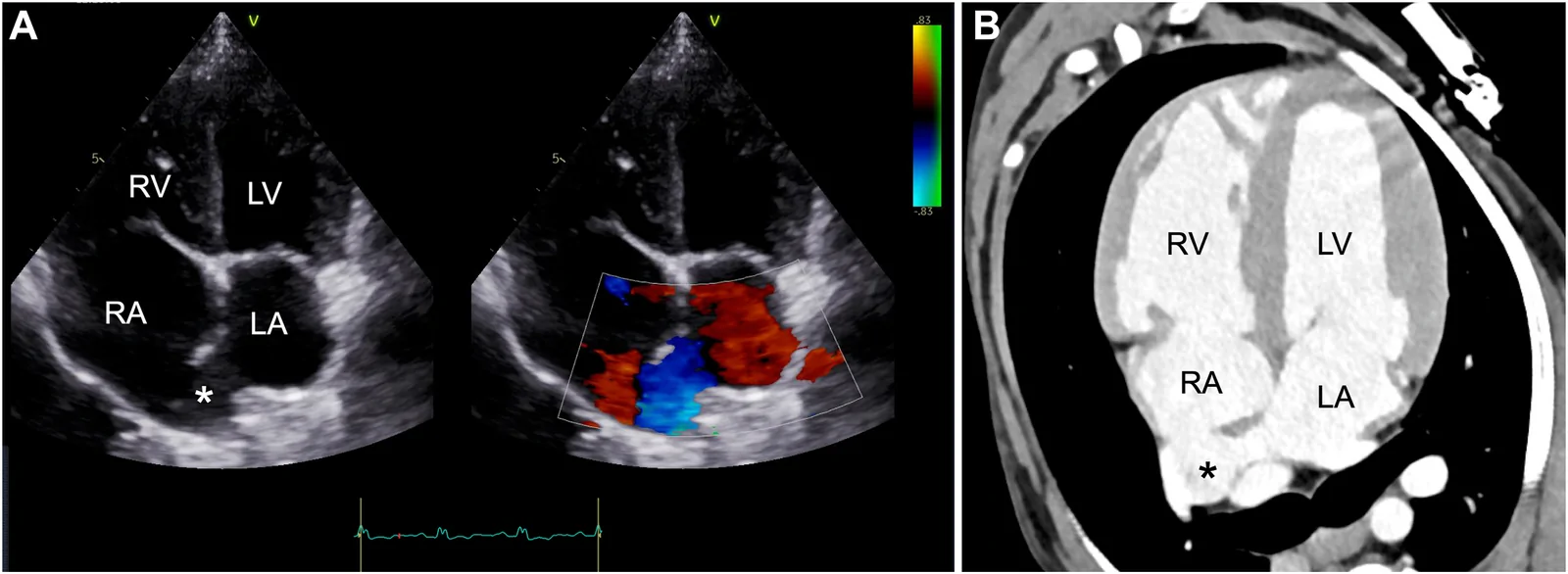
Two-dimensional TTE, left apical 4-chamber view **(A)** and the corresponding CT angiography image **(B),** demonstrates the sinus venosus defect. Simultaneous CFD suggests a potential distal connection between the right cranial and/or right middle lobe PVein (∗) and the dorsal aspect of the RA, with possible flow across the connection. Computed tomography angiography confirms a connection between the right middle lobe PVein (∗) and the roof of the RA. *LV*, Left ventricle; *RV*, right ventricle.

Although the murmur was attributed to PV stenosis, the degree of right atrial and right ventricular dilation appeared disproportionate to the severity of obstruction. The mild right ventricular wall thickening and modest pulmonary outflow pressure gradient supported only mild stenosis (and pulmonary outflow velocity could be artificially overestimated in the presence of a left-to-right shunt). The mild tricuspid and trace pulmonary regurgitation likewise seemed insufficient to explain the chamber dilation. Collectively, these findings raised suspicion for a left-to-right shunt; left atrial flow reversal suggested drainage into the RA or a systemic vein via an extracardiac shunt, although a concurrent intracardiac shunt could not be ruled out. However, the pulmonary-to-systemic flow ratio (*Q*_*p*_/*Q*_*s*_) could not be calculated to confirm the presence of a shunt because the concurrent valvular stenosis and regurgitation precluded accurate measurements.

The dog returned 1 month later for CT of the head and neck, and thoracic CT angiography was also elected for further investigation. Images were acquired in contiguous transverse helical slices and reconstructed into 1 mm volumes. Bolus tracking was performed (1 mL/kg iohexol administered via power injector at 2.5 mL/s) with a region of interest placed over the caudal vena cava, and scanning was initiated after reaching an attenuation threshold of 80 Hounsfield units. On review, the right middle lobe PVein remained connected to the LA but also exhibited an abnormal communication with the dorsocaudal aspect of the RA (Figures 5B and 6, Video 4). Two right cranial lobe PVeins, however, drained exclusively into the RA at the same confluence (Figure 7). The right caudal lobe, accessory lobe, and all left-sided PVeins connected normally to the LA. The interatrial septum appeared intact at locations where ostium secundum and ostium primum defects are expected, including the regions of the fossa ovalis and atrioventricular valve insertion. Other findings corroborated the TTE, including focal narrowing at the level of the PV, moderate dilation of the main pulmonary artery, and moderate dilation of the RA and RV.

**Figure 6:**
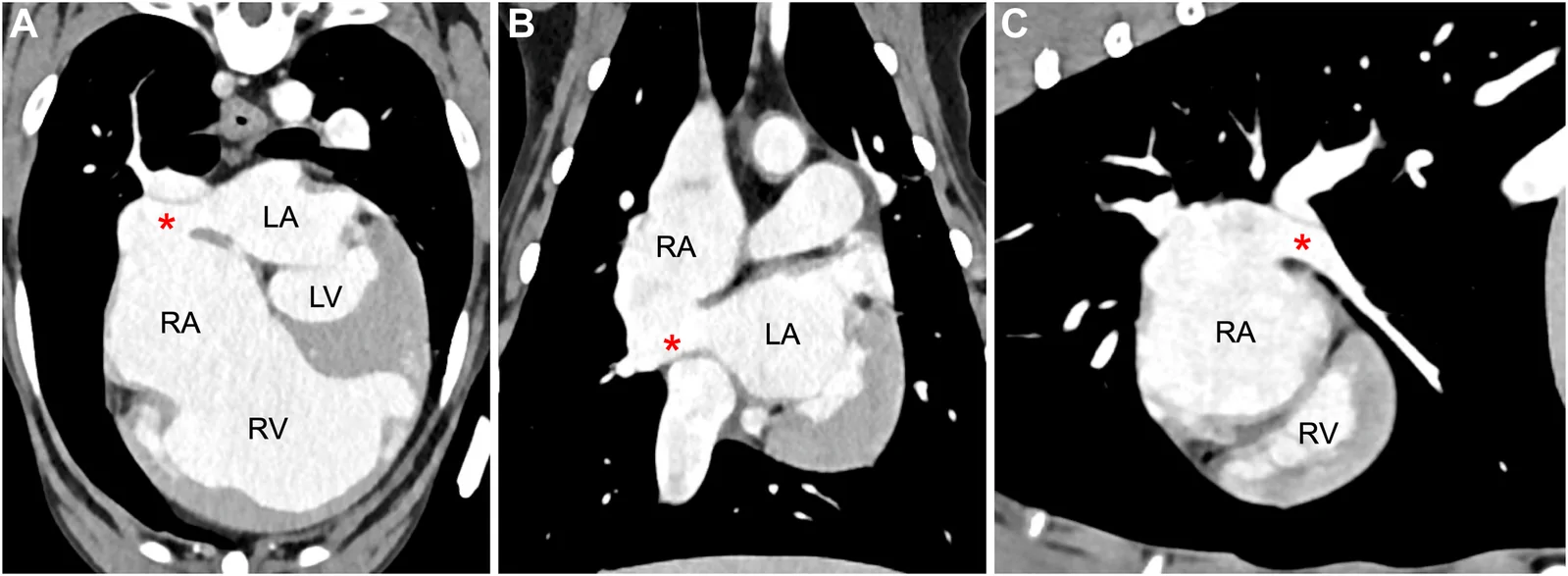
Computed tomography angiography, transverse **(A)**, dorsal **(B)**, and sagittal planes **(C)**, demonstrates PAPVD consistent with a sinus venosus defect. This manifests as a shunting window between the right middle lobe PVein (∗) and the RA, while the PVein remains connected to the LA. Moderate dilation of the RA and RV is present. *LV*, Left ventricle; *RV*, right ventricle.

**Figure 7:**
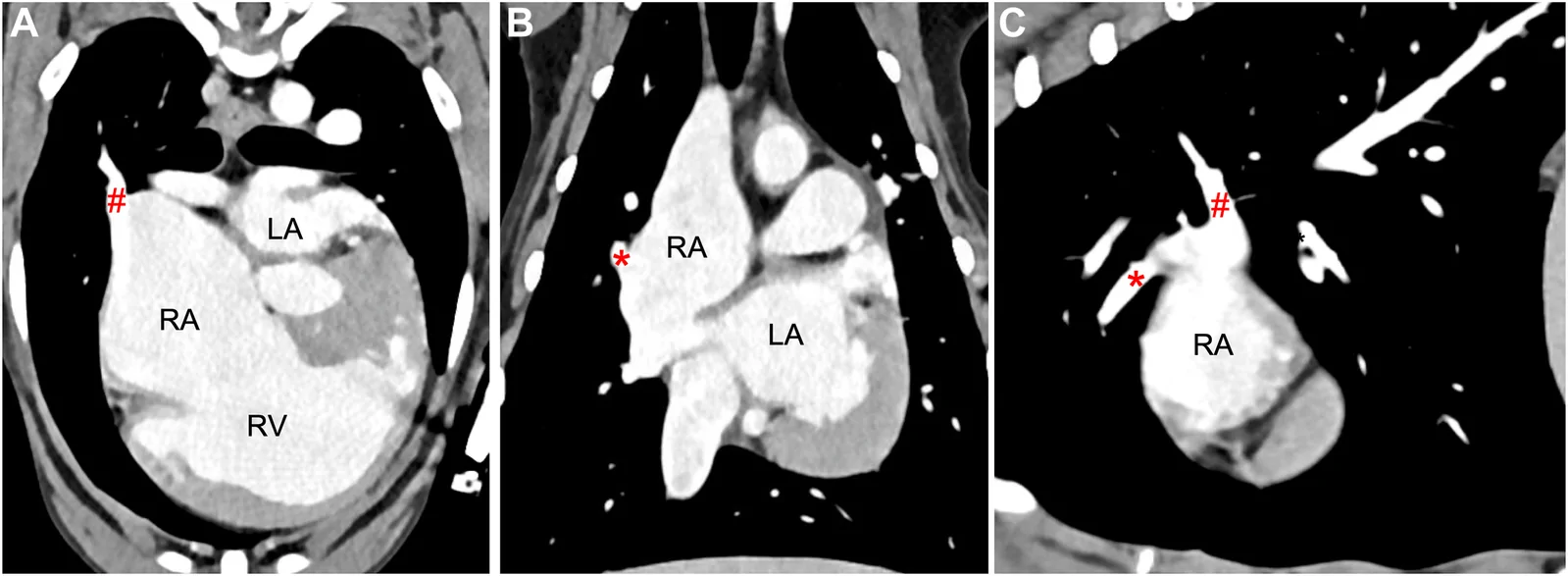
Computed tomography angiography, transverse **(A)**, dorsal **(B)**, and sagittal planes **(C)**, demonstrates additional PAPVCs involving 2 PVeins. Two right cranial lobe PVeins (∗, ^#^) connect exclusively to the RA, near the same confluence as the right middle lobe PVein. *RV*, Right ventricle.

Computed tomography of the head revealed otitis externa and unaffected bullae in the right ear. Video otoscopy identified a black foreign body and a ruptured tympanic membrane. The foreign body was promptly removed, and the ear canal and bullae were thoroughly flushed. The consulting cardiologist was not on duty at the time of the CT, so repeat TTE was not pursued. The patient recovered uneventfully from anesthesia and was discharged from the hospital with topical ear medications. After discussing medical and surgical treatment options for the cardiac abnormalities, the owners elected routine annual monitoring given the absence of clinical signs, lack of proven medical therapy, and limited access to open-heart surgery. While atenolol was considered for the PV stenosis, the perceived mild severity of the lesion precluded the need for medical therapy.

## Discussion

Although PAPVC and PAPVD have distinct definitions, this dog exhibited features of both in a pattern consistent with a sinus venosus defect. Unlike previously reported veterinary cases, initial echocardiographic suspicion was primarily driven by left atrial flow reversal within the right middle lobe PVein, suggesting anomalous drainage into a lower-pressure systemic venous structure.

While a common differential for extracardiac left-to-right shunts, PAPVC was not initially considered in this case because it alone could not explain the observed left atrial flow reversal. Anomalous pulmonary venous connections typically result from early atresia of the common PVein, which severs the connection to the LA, while pulmonary-systemic venous connections persist.^1^^,^^2^ During the early stages of development, the lungs are enmeshed by the splanchnic (foregut) vascular plexus, a portion of which differentiates into the pulmonary vascular bed. The common PVein then forms as an evagination from the heart and establishes a connection with the pulmonary venous plexus, after which the pulmonary-splanchnic venous connections normally involute. The common PVein is subsequently incorporated into the LA, leaving the PVeins to connect directly to the LA. If the common PVein fails to develop or becomes atretic early in its development, this can lead to persistence and enlargement of the collateral channels between the pulmonary and splanchnic venous plexuses, resulting in total anomalous pulmonary venous connections. If only the right or left portion of the common PVein becomes atretic, then PAPVC would occur. Interestingly, most cases of PAPVC involve right-sided PVeins in humans and in dogs.^1^^,^^3^, ^4^, ^5^ The connections can be cardiac, with drainage to the RA, or extracardiac, with drainage to systemic veins such as the cranial or caudal vena cava or their tributaries.

Only PAPVD provides an extracardiac connection between the LA and the systemic venous system and is most commonly associated with the sinus venosus defect, a diagnosis consistent with the findings in this dog.^1^ This defect allows shunting at the atrial or supra-atrial level (dorsal to the atrial roof) and is often classified under atrial septal defects, along with ostium primum, ostium secundum, and unroofed coronary sinus defects. However, they do not involve the fossa ovalis, septum primum, or septum secundum. In humans, the underlying defect is historically described as a deficiency of the muscular common wall between the right superior vena cava and the right upper PVein or between the RA and the right upper and lower PVeins.^1^^,^^6^ This results in unroofing of the right upper PVein and its branches into the right superior cranial vena cava (sinus venosus of the superior vena cava type) or unroofing of the right upper and lower PVeins into the RA (sinus venosus defect of the RA type), as observed in this dog. This defect is traditionally thought to involve the sinus venosus part of the RA and the common pulmonary venous part of the LA and is thus better considered as a veno-venous malformation than a true atrial septal defect.^6^

Despite the distinction between anomalous connection and anomalous drainage, most sinus venosus defects resemble this case in being accompanied by PAPVC, which occurs in more than 90% of cases in humans.^7^ Although historically not described as sinus venosus defects, most contemporary definitions include instances in which the orifice of the systemic vein receiving the anomalous PVein(s) overrides the rims of the fossa ovalis, sparing the true atrial septum and creating biatrial connection of the caval vein.^8^ However, no universally accepted definition exists, a limitation compounded by variability among the diagnostic imaging modalities used to investigate the anatomy. Sinus venous defects are rarely reported in dogs; however, among 3 previous case reports and a retrospective study of 10 dogs, all patients had concurrent PAPVC documented by CT angiography.^9^, ^10^, ^11^, ^12^ In all dogs, either the right cranial lobe PVeins alone or both the right cranial and right middle lobe PVeins inserted into the RA, cranial vena cava, or their junction without connecting to the LA. As in this dog and in humans, their main shunts appeared to override the fossa ovalis. These findings suggest that the sinus venosus defect may represent a variant of PAPVC.

This dog was diagnosed with a sinus venosus defect and is unique among reported veterinary cases in that the defect, later confirmed by CT angiography, was inferred indirectly from left atrial flow reversal rather than visualized directly on TTE. The defect was identified solely due to routine positioning of CFD, unrelated to the assumed areas of interest, during a comprehensive echocardiographic examination. The same circumstance was also reported in a human undergoing transesophageal echocardiography for suspected tachycardia-induced cardiomyopathy secondary to atrial flutter.^13^ Recognition also required careful interpretation to avoid attributing all observed hemodynamic and chamber remodeling changes solely to concurrent PV stenosis and tricuspid regurgitation, which are far more common conditions to diagnose in dogs. Notably, CFD findings were inconclusive for flow into the RA across the defect, likely due to the low shunting velocity and proximity to caval flow; further evaluation with an agitated saline study and/or transesophageal echocardiography may have enhanced diagnostic confidence.^14^ Nevertheless, this case highlights the importance of performing a complete, systematic echocardiographic examination and maintaining a high index of suspicion for concurrent or uncommon congenital anomalies, even when an apparent primary lesion is present.

Regardless of terminology, both anomalous connection and anomalous drainage result in a left-to-right shunt, leading to right-sided volume overload.^1^ Many cases are ultimately identified during evaluation for unexplained right-sided cardiac enlargement. Most affected dogs are asymptomatic or are presented with clinical signs such as exercise intolerance, syncope, and dyspnea.^3^^,^^4^^,^^9^, ^10^, ^11^, ^12^ Increased pulmonary blood flow from shunting can result in pulmonary vascular remodeling and increased pulmonary vascular resistance, potentially progressing to pulmonary arterial hypertension.^1^^,^^15^ Surgical correction is the primary treatment option, although the approach varies according to the underlying anatomy.^1^^,^^8^ Successful patch closure of the sinus venosus defect and accompanying PAPVC has been reported in dogs in Japan and South Korea.^10^, ^11^, ^12^

## Conclusion

This case documents a dog with PAPVD initially suggested by left atrial flow reversal on TTE and later confirmed on CT angiography as a sinus venosus defect with concurrent PAPVC. It emphasizes the value of comprehensive echocardiographic evaluation, including systematic use of CFD, and underscores the importance of avoiding diagnostic anchoring in the presence of concurrent lesions.

## Ethics Statement

The authors declare that this report involves a client-owned dog receiving standard-of-care veterinary services in accordance with the Principles of Veterinary Medical Ethics of the American Veterinary Medical Association and does not involve a laboratory research animal undergoing experimental procedures.

## Consent Statement

The authors declare that informed patient consent was not provided because this report is based on retrospectively reviewed medical records and diagnostic images from a client-owned dog receiving owner-approved veterinary care and contains no identifying information regarding the owner or animal.

## Funding

The authors declare that this report did not receive any specific grant from funding agencies in the public, commercial, or not-for-profit sectors.

## Disclosure Statement

The authors reported no actual or potential conflicts of interest relative to this document.

## Supplementary Data

Video 1 Two-dimensional TTE, right parasternal long-axis 4-chamber view, demonstrates moderate dilation of the RA and RV, with only mild right ventricular wall thickening. *RV*, Right ventricle.

Video 2 Two-dimensional TTE, left apical 4-chamber view with simultaneous color-flow Doppler, demonstrates continuous pulmonary venous flow away from the LA into the right cranial and/or right middle lobe PVein.

Video 3 Two-dimensional TTE, left apical 4-chamber view with simultaneous color-flow Doppler, demonstrates a potential distal connection between the right cranial and/or right middle lobe PVein and the dorsal aspect of the RA. There is possible flow across the connection; however, it is not as continuous or convincing as the left atrial flow reversal and is therefore not definitive.

Video 4 Computed tomography angiography sequence, displayed from cranial to caudal, demonstrates the anatomical relationships of the PVeins, RA, LA, and surrounding structures.

## References

[1] Brown D.W., Geva T., Shaddy R.E., Penny D.J., Feltes T.F., Cetta F., Mital S. (2022). Moss and Adams’ Heart Disease in Infants, Children, and Adolescents.

[2] Douglas Y.L., Jongbloed M.R., Deruiter M.C., Gittenberger-de Groot A.C. (2011). Normal and abnormal development of pulmonary veins: state of the art and correlation with clinical entities. Int J Cardiol.

[3] Fujii Y., Ishikawa T., Sunahara H., Sugimoto K., Kanai E., Kayanuma H. (2014). Partial anomalous pulmonary venous connection in 2 Miniature Schnauzers. J Vet Intern Med.

[4] Thorn C.L., Ford N.R., Sleeper M.M. (2017). Partial anomalous pulmonary venous connection in a dog. J Vet Cardiol.

[5] Verma A.K., Sethi S., Kohli N. (2022). Partial anomalous pulmonary venous connection: state-of-the-art review with assessment using a multidetector computed tomography angiography. Pol J Radiol.

[6] Van Praagh S., Carrera M.E., Sanders S.P., Mayer J.E., Van Praagh R. (1994). Sinus venosus defects: unroofing of the right pulmonary veins—anatomic and echocardiographic findings and surgical treatment. Am Heart J.

[7] Attenhofer Jost C.H., Connolly H.M., Danielson G.K., Bailey K.R., Schaff H.V., Shen W.K. (2005). Sinus venosus atrial septal defect: long-term postoperative outcome for 115 patients. Circulation.

[8] Chowdhury U.K., Anderson R.H., Pandey N.N., Sharma S., Sankhyan L.K., George N. (2022). A reappraisal of the sinus venosus defect. Eur J Cardiothorac Surg.

[9] Jeraj K., Ogburn P.N., Johnston G.R., Edwards W., Yano B., Brunson D. (1980). Atrial septal defect (sinus venosus type) in a dog. J Am Vet Med Assoc.

[10] Mizuno T., Mizuno M., Harada K., Takano H., Shinoda A., Takahashi A. (2020). Surgical correction for sinus venosus atrial septal defect with partial anomalous pulmonary venous connection in a dog. J Vet Cardiol.

[11] Kim K.M., Moon C.H., Lee W.J., Kim W.J., Kim M., Jeong J. (2024). Surgical correction of a sinus venosus atrial septal defect with partial anomalous pulmonary venous connections using cardiac computed tomography imaging and a 3D-printed model. Animals (Basel).

[12] Chen A., Kurogochi K., Ono S., Uechi M. (2025). Clinical characteristics, echocardiographic findings, and computed tomography angiography in the diagnosis of sinus venosus atrial septal defect with partial anomalous pulmonary venous connection in 10 dogs. J Vet Cardiol.

[13] Baig M., Nasir A., Ou J. (2024). Unusual Doppler in pulmonary veins leads to the diagnosis of unusual partial anomalous pulmonary venous return with dual connections. Ann Case Rep.

[14] Ammash N.M., Seward J.B., Warnes C.A., Connolly H.M., O'Leary P.W., Danielson G.K. (1997). Partial anomalous pulmonary venous connection: diagnosis by transesophageal echocardiography. J Am Coll Cardiol.

[15] Vogel M., Berger F., Kramer A., Alexi-Meshkishvili V., Lange P.E. (1999). Incidence of secondary pulmonary hypertension in adults with atrial septal or sinus venosus defects. Heart.

